# Scarless genome editing technology and its application to crop improvement

**DOI:** 10.1270/jsbbs.23045

**Published:** 2024-03-09

**Authors:** Kazuya Ikeda

**Affiliations:** 1 Bayspair Inc., 319 Bernardo Avenue, Mountain View, CA 94043, USA

**Keywords:** genome editing, GWAS, scarless editing

## Abstract

The advent of CRISPR/Cas9 has had a disruptive impact on the world by bringing about dramatic progress and rapid penetration of genome editing technology. However, even though gene disruption can be easily achieved, there has been a challenge in freely changing the sequence. To solve this problem, various novel technologies have emerged in recent years to realize free rewriting of genome sequences. In this review, scarless editing by two-step HDR, a technology that can freely rewrite genomes from a single nucleotide to more than several thousand nucleotides, will be introduced.

## Current status of the use of genome editing technology in plant science

A decade ago, genome editing using CRISPR/Cas9 in eukaryotes was demonstrated (Cho *et al.* 2013, Cong *et al.* 2013, Feng *et al.* 2013, Jinek *et al.* 2013, Mali *et al.* 2013), and now the technology has penetrated far and wide into the scientific community, with numerous studies underway (Wang and Doudna 2023). In the field of plant science, genome editing technology has been used not only for research on plant pathophysiology but also for practical breeding (Nerkar *et al.* 2022), and some genome-edited crops have already been commercialized and are being consumed by humans (Waltz 2022). Thus, genome editing is not an experimental and uncommon technology handled only by researchers, but a technology that has entered a phase of implementation in the public. By contrast, what this genome editing technology, which includes the nuance of free rewriting of the genomic sequence, truly can do without difficulty is to destroy genes. In fact, majority of research outputs using genome editing (Matres *et al.* 2021) and the genome-edited crops under development (Nagamine and Ezura 2022, Xu *et al.* 2020) are the result of gene disruption. Since it is possible to develop useful varieties by specifically destroying genes that have adverse effects on breed characteristics, genome editing technology is a revolutionary technology that can achieve this with high efficiency and rapidity. On the other hand, Genome-wide association studies (GWAS) suggest that majority of genetic variation that determines quantitative traits or relates disease is not comprised of gene deletions or amino acid substitutions, but rather copy number variations and SNPs in regulatory regions of gene expression such as promoter or non-coding regions in not only in human (Alsheikh *et al.* 2022, Hindorff *et al.* 2009) but also plants such as maize (Wallace *et al.* 2014). Identified sequence variation can be introduced by crossbreeding, but due to linkage disequilibrium, several megabases of surrounding sequences are introduced simultaneously, making high-resolution analysis difficult in physiological studies, and it requires several generations of backcrossing to reduce risk of linkage drag which may cause problems in variety improvement (Epstein *et al.* 2023). Therefore, it would be ideal if only the causal variant could be made by genome rewriting technology, but such sequence alteration is challenging by conventional genome editing methods.

## Genome rewriting difficulties and editing scar

**Fig. 1.**
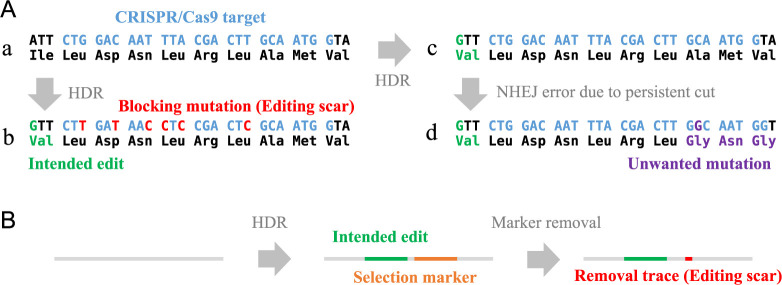
Editing scars in HDR based editing. A. Synonymous substitutions additionally introduced in HDR based genome editing to change amino acid sequence coded in genome. If the purpose of genome editing is to replace the initial isoleucine with valine, the genomic sequence (a) needs to be edited as in (b) to prevent continued cleavage by CRISPR/Cas9 after the HDR event. In this design, editing scars are synonymous mutations named as blocking mutation which are introduced to change the sequence of CRISPR/Cas9 target. If no synonymous substitutions are introduced as in (c), the CRISPR/Cas9 target remains after HDR, so it continues to undergo CRISPR/Cas9 cleavage and eventually the sequence is randomly altered by NHEJ error or MMEJ, resulting in unintended insertions or deletions, as in (d). B. Sequences remaining after removal of selection markers. Often selective markers are used to overcome the low HDR efficiency. Selection markers are unnecessary sequences for the final editing product and are therefore removed by the Cre/loxP or piggyBac systems. However, in the case of Cre/loxP, the loxP sequence remains, and in the piggyBac system, the sequence TTAA remains.

To rewrite a genome to a desired sequence using CRISPR/Cas9, the genome editing must be mediated by a mechanism called homology directed repair (HDR) but not non-homologous end joining (NHEJ) nor microhomology-mediated end joining (MMEJ) which cause gene destruction (Porteus 2016). However, genome editing via HDR is not easy due to its low efficiency and the limitations of the editing design that leaves editing scars. The efficiency of HDR-mediated genome editing is usually less than one-tenth to one-hundredth of the frequency of gene disruption via NHEJ or MMEJ, and the cells edited as intended by HDR are often less than 1% of the cell population that receives editing (Kim *et al.* 2022, Luo *et al.* 2023). In this case, screening of more than several hundred clones is required to obtain cells with intended editing. Editing scar means additional sequence changes to the CRISPR/Cas9 target sequence that must be intentionally introduced to prevent CRISPR/Cas9 from continuing to cut the target site after the targeted sequence change by HDR, resulting in gene disruption via NHEJ or MMEJ (Fig. 1A), or unwanted sequences left after and removal of selection markers by such as Cre/loxP system when the use of selection markers is unavoidable to obtain correctly edited cells due to low HDR efficiency (Fig. 1B). CRISPR/Cas9 has mismatch tolerance (Hsu *et al.* 2013), and even for Cas9 mutants with improved fidelity, it is difficult to avoid re-cutting and introducing undesired mutations by substituting a single nucleotide other than the PAM sequence which is critical for sequence recognition by Cas9 (Kim *et al.* 2023). In addition, the efficiency of introducing the intended edit by HDR decreases exponentially depending on the distance between the editing position and cutting position (cut-to-edit distance) which is determined by guide RNA sequence (Paquet *et al.* 2016). Therefore, single nucleotide substitutions face multiple challenges unless the edit is on the PAM or very near the cut. Therefore, the cases in which CRISPR/Cas9 has succeeded in making single nucleotide substitutions, the variants detected by GWAS, without leaving editing scars are limited to substitutions in the PAM sequence or mutations very close to the cleavage site. Base editing (Komor *et al.* 2016) and Prime Editing (Anzalone *et al.* 2019) have emerged as other means of genome rewriting. While these are very attractive tools and available in plant cells (Jiang *et al.* 2020, Li *et al.* 2020), base editing is limited in where it can edit (Jeong *et al.* 2020), and prime editing remains inefficient and requires complex design and optimization (Hassan *et al.* 2020). Also, these tools can only edit where the guide RNA can be designed. To edit specific genomic locations, guide RNAs must be designed to target unique sequences. In species with complex genomes, such as maize, where nearly 85% of the genome contains highly repetitive sequences (Schnable *et al.* 2009), and bread wheat, a hexaploid, which is rich in similar sequences (Zimin *et al.* 2017), it is rarely possible to specify a single genetic region with a sequence of about 20 or so bases recognized by a designed guide RNA. In such situations, it is often impossible to design an appropriate guide RNA for the area to be edited.

## Why scarless editing is important?

If the change in the amino acid sequence encoded by the gene of interest is for research or breeding purposes, editing scars may be allowed as synonymous substitutions that do not change the amino acid sequence or by placing scars in untranslated regions (Kim *et al.* 2022). However, the non-random and biased use of synonymous codons during evolution in many organisms (Chiapello *et al.* 1998, Hershberg and Petrov 2008) suggests that synonymous substitutions are not always functionally synonymous. In fact, it is known that synonymous substitutions and mutations in introns that do not change the amino acid sequence affect splicing (Sarkar *et al.* 2022), mRNA stability, protein translation kinetics, and also significantly affect the three-dimensional structure of proteins (Brule and Grayhack 2017) in eukaryotes. Reflecting these experimental facts, numerous genetic diseases have been shown to be caused by synonymous substitutions in humans (Bali and Bebok 2015). Therefore, it is very difficult to predict how an editing scar will affect function even if the amino acid sequence is not changed. Furthermore, when the purpose is to modify the sequence in the region responsible for regulation of gene expression, which is believed to determine many characteristics of cultivars, the introduction of editing scars is basically unacceptable since there is no concept of synonymous substitution. Hence, ideally, an editing method should be used that does not leave scars.

## Scarless editing by two-step HDR

**Fig. 2.**
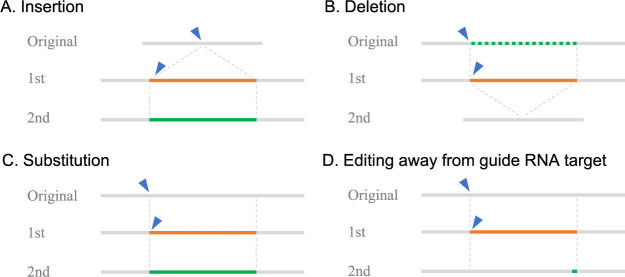
Two-step HDR editing variations combined with marker selection. Blue arrowhead: CRISPR/Cas9 target, orange line: selection marker expression cassette, green line: intended edit, green dotted line; sequence to be removed.

**Fig. 3.**
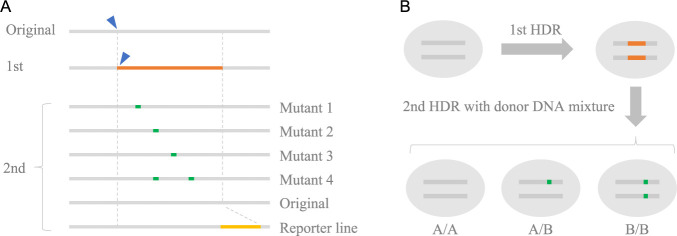
Variations of genotypes that can be created in the second HDR step. Blue arrowhead: CRISPR/Cas9 target, orange line: selection marker expression cassette, green line: intended edit, yellow line: reporter gene such as fluorescence or luminescence.

To meet the demand for genome editing without leaving editing scars as described above, two-step HDR has been developed (Paquet *et al.* 2016). In this strategy, to achieve a single nucleotide substitution, the first HDR changes the sequence of multiple bases, including the editing scar, and the second HDR leaves the desired single nucleotide substitution and restores the other changed bases to remove the editing scars. This strategy enables circumvent of difficult single nucleotide substitutions. However, this method does not solve the problem of low HDR efficiency. In the case of human iPS cells for which this method was developed, screening of 400–600 clones is recommended at each stage (Kwart *et al.* 2017), and since HDR efficiency is similarly low in plants, genome editing with this method is expected to be a very laborious task. Thereafter, to overcome the low HDR efficiency, a method combining two-step HDR and positive-negative selection with selection markers was developed (Ikeda *et al.* 2018). In this method, in the first step, the region containing cut-to-edit is replaced with a selection marker expression cassette, and genome-edited cells are enriched by positive selection. Then, in the second step, the selection marker expression cassette is replaced with a desired sequence, and cells that do not express the selection marker are enriched by negative selection. The use of selection markers relieves the pressure to achieve a highly efficient HDR and allows us to freely make just a single base substitution or more than thousands of base insertions, substitutions, and deletions in the genomic region replaced by the selection marker at the first step without screening a large number of clones (Fig. 2A–2C). In addition, it enabled editing at loci away from the guide RNA target, which was not possible with CRISPR/Cas9 or its derivative technologies (Fig. 2D). Thus, the requirement to design an appropriate guide RNA on the editing site has been eliminated, and the greater choice of guide RNAs that can be used allows for the use of guide RNAs with low off-target risk. Although this method is similar to the method traditionally employed to introduce large sequences by introducing the intended editing along with selection markers and then removing the selection markers with a Cre/loxP system or other system, several significant advantages over the conventional method can be noted. First, two-step HDR leaves no traces of selection marker removal such as loxP sequences. Furthermore, the feature of introducing only the selection marker at the first step and the target sequence at the second step allows variations to be created at the second editing step. This feature can be used to introduce reporter genes or various combinations of genotypes for SNPs scattered over more than kilobases at once (Fig. 3A). Additionally, when the selection markers are introduced into the bi-allelic in the first step, two donor vectors, e.g. genotype A and genotype B, can be used in the second step to produce AA, AB, and BB genotypes simultaneously (Fig. 3B). At this time, there are no reported cases of this method being used in plants. However, this method is consisted by a simple procedure that repeats twice the insertion or removal of a selection marker through HDR and marker selection. Since it has been reported that HDR-mediated insertion of a selection marker, which corresponds to the first step of this method, is already feasible in plants (Miki *et al.* 2018, Svitashev *et al.* 2015, Zhang *et al.* 2023), this scarless editing method would also be feasible in plants by using appropriate selection markers.

## Future perspectives

Through GWAS analysis, many genomic sequence variations have been identified that can explain simple traits such as metabolic profile change (Wallace *et al.* 2014) and complex quantitative traits such as plant height (Peiffer *et al.* 2014), abiotic stress tolerance (Chen *et al.* 2017, Javid *et al.* 2022, Yasir *et al.* 2019) and disease tolerance (Kump *et al.* 2011, Poland *et al.* 2011). However, the genetic changes detected by GWAS may not be the true causal mutations as they may be mutations that do not affect function that are linked to the true causal mutations (Tam *et al.* 2019). In addition, the mechanisms by which such genome sequence variation affect function remain a black box. While a number of strategies, including statistical methods and genomic functional annotation (Broekema *et al.* 2020, Cannon and Mohlke 2018, Schaid *et al.* 2018), have been widely applied to prioritize causal variants (called fine mapping) and their target genes, definitive mechanism identification requires direct functional analysis studies comparing the presence or absence of the relevant mutations. Here, the fact that the majority of mutations detected by GWAS are synonymous mutations that do not result in amino acid sequence changes or are found in untranslated or intergenic regions makes it difficult to verify the causal variants and their targets and to elucidate the molecular mechanisms. If the functional change is due to amino acid sequence substitution, it is relatively easy to infer that the function of the protein in question is affected, and there are many options for functional analysis using artificial protein expression systems. However, the effects of the other type of genomic changes on physiological phenotypes are expected to involve various mechanisms, such as epigenetic mechanisms, transcriptional regulators, mRNA stability and splicing, translation regulation, and regulation by non-coding RNAs, and the evaluation systems are complex, making it difficult even for human research, which is considered the most advanced genome analysis (Rao *et al.* 2021). The ultimate solution to this problem is scarless editing, which enables the identification of genetic changes that have beneficial effects on cultivar traits and their mechanisms by comparing isogenic plants with only the intended mutations. Finally, the useful genetic changes thus identified can be introduced into other cultivars quickly by using scarless editing.

## Author Contribution Statement

K.I. wrote the manuscript.

## References

[B1] Alsheikh, A.J., S. Wollenhaupt, E.A. King, J. Reeb, S. Ghosh, L.R. Stolzenburg, S. Tamim, J. Lazar, J.W. Davis and H.J. Jacob (2022) The landscape of GWAS validation; systematic review identifying 309 validated non-coding variants across 130 human diseases. BMC Med Genomics 15: 74. 35365203 10.1186/s12920-022-01216-w PMC8973751

[B2] Anzalone, A.V., P.B. Randolph, J.R. Davis, A.A. Sousa, L.W. Koblan, J.M. Levy, P.J. Chen, C. Wilson, G.A. Newby, A. Raguram et al. (2019) Search-and-replace genome editing without double-strand breaks or donor DNA. Nature 576: 149–157. 10.1038/s41586-019-1711-4 PMC6907074 31634902

[B3] Bali, V. and Z. Bebok (2015) Decoding mechanisms by which silent codon changes influence protein biogenesis and function. Int J Biochem Cell Biol 64: 58–74. 25817479 10.1016/j.biocel.2015.03.011 PMC4461553

[B4] Broekema, R.V., O.B. Bakker and I.H. Jonkers (2020) A practical view of fine-mapping and gene prioritization in the post-genome-wide association era. Open Biol 10: 190221. 31937202 10.1098/rsob.190221 PMC7014684

[B5] Brule, C.E. and E.J. Grayhack (2017) Synonymous codons: choose wisely for expression. Trends Genet 33: 283–297. 28292534 10.1016/j.tig.2017.02.001 PMC5409834

[B6] Cannon, M.E. and K.L. Mohlke (2018) Deciphering the emerging complexities of molecular mechanisms at GWAS loci. Am J Hum Genet 103: 637–653. 30388398 10.1016/j.ajhg.2018.10.001 PMC6218604

[B7] Chen, J., R. Chopra, C. Hayes, G. Morris, S. Marla, J. Burke, Z. Xin and G. Burow (2017) Genome-wide association study of developing leaves’ heat tolerance during vegetative growth stages in a sorghum association panel. Plant Genome 10: 2. 10.3835/plantgenome2016.09.0091 28724078

[B8] Chiapello, H., F. Lisacek, M. Caboche and A. Hénaut (1998) Codon usage and gene function are related in sequences of *Arabidopsis thaliana*. Gene 209: GC1–GC38. 9583944 10.1016/s0378-1119(97)00671-9

[B9] Cho, S.W., S. Kim, J.M. Kim and J.-S. Kim (2013) Targeted genome engineering in human cells with the Cas9 RNA-guided endonuclease. Nat Biotechnol 31: 230–232. 23360966 10.1038/nbt.2507

[B10] Cong, L., F.A. Ran, D. Cox, S. Lin, R. Barretto, N. Habib, P.D. Hsu, X. Wu, W. Jiang, L.A. Marraffini et al. (2013) Multiplex genome engineering using CRISPR/Cas systems. Science 339: 819–823. 23287718 10.1126/science.1231143 PMC3795411

[B11] Epstein, R., N. Sajai, M. Zelkowski, A. Zhou, K.R. Robbins and W.P. Pawlowski (2023) Exploring impact of recombination landscapes on breeding outcomes. Proc Natl Acad Sci USA 120: e2205785119. 36972450 10.1073/pnas.2205785119 PMC10083619

[B12] Feng, Z., B. Zhang, W. Ding, X. Liu, D.-L. Yang, P. Wei, F. Cao, S. Zhu, F. Zhang, Y. Mao et al. (2013) Efficient genome editing in plants using a CRISPR/Cas system. Cell Res 23: 1229–1232. 23958582 10.1038/cr.2013.114 PMC3790235

[B13] Hassan, M.M., G. Yuan, J.-G. Chen, G.A. Tuskan and X. Yang (2020) Prime editing technology and its prospects for future applications in plant biology research. Biodes Res 2020: 9350905. 37849904 10.34133/2020/9350905 PMC10530660

[B14] Hershberg, R. and D.A. Petrov (2008) Selection on codon bias. Annu Rev Genet 42: 287–299. 18983258 10.1146/annurev.genet.42.110807.091442

[B15] Hindorff, L.A., P. Sethupathy, H.A. Junkins, E.M. Ramos, J.P. Mehta, F.S. Collins and T.A. Manolio (2009) Potential etiologic and functional implications of genome-wide association loci for human diseases and traits. Proc Natl Acad Sci USA 106: 9362–9367. 19474294 10.1073/pnas.0903103106 PMC2687147

[B16] Hsu, P.D., D.A. Scott, J.A. Weinstein, F.A. Ran, S. Konermann, V. Agarwala, Y. Li, E.J. Fine, X. Wu, O. Shalem et al. (2013) DNA targeting specificity of RNA-guided Cas9 nucleases. Nat Biotechnol 31: 827–832. 23873081 10.1038/nbt.2647 PMC3969858

[B17] Ikeda, K., N. Uchida, T. Nishimura, J. White, R.M. Martin, H. Nakauchi, V. Sebastiano, K.I. Weinberg and M.H. Porteus (2018) Efficient scarless genome editing in human pluripotent stem cells. Nat Methods 15: 1045–1047. 30504872 10.1038/s41592-018-0212-y

[B18] Javid, S., M.R. Bihamta, M. Omidi, A.R. Abbasi, H. Alipour and P.K. Ingvarsson (2022) Genome-Wide Association Study (GWAS) and genome prediction of seedling salt tolerance in bread wheat (*Triticum aestivum* L.). BMC Plant Biol 22: 581. 36513980 10.1186/s12870-022-03936-8 PMC9746167

[B19] Jeong, Y.K., B. Song and S. Bae (2020) Current status and challenges of DNA base editing tools. Mol Ther 28: 1938–1952. 32763143 10.1016/j.ymthe.2020.07.021 PMC7474268

[B20] Jiang, Y.-Y., Y.-P. Chai, M.-H. Lu, X.-L. Han, Q. Lin, Y. Zhang, Q. Zhang, Y. Zhou, X.-C. Wang, C. Gao et al. (2020) Prime editing efficiently generates W542L and S621I double mutations in two ALS genes in maize. Genome Biol 21: 257. 33023639 10.1186/s13059-020-02170-5 PMC7541250

[B21] Jinek, M., A. East, A. Cheng, S. Lin, E. Ma and J. Doudna (2013) RNA-programmed genome editing in human cells. Elife 2: 00471. 10.7554/eLife.00471 PMC3557905 23386978

[B22] Kim, J.H., J. Yu, H.K. Kim, J.Y. Kim, M.-S. Kim, Y.-G. Cho, S. Bae, K.K. Kang and Y.J. Jung (2022) Genome editing of golden SNP-carrying lycopene epsilon-cyclase (*LcyE*) gene using the CRSPR-Cas9/HDR and geminiviral replicon system in rice. Int J Mol Sci 23: 10383. 36142294 10.3390/ijms231810383 PMC9499184

[B23] Kim, Y.-H., N. Kim, I. Okafor, S. Choi, S. Min, J. Lee, S.-M. Bae, K. Choi, J. Choi, V. Harihar et al. (2023) Sniper2L is a high-fidelity Cas9 variant with high activity. Nat Chem Biol 19: 972–980. 36894722 10.1038/s41589-023-01279-5 PMC10374439

[B24] Komor, A.C., Y.B. Kim, M.S. Packer, J.A. Zuris and D.R. Liu (2016) Programmable editing of a target base in genomic DNA without double-stranded DNA cleavage. Nature 533: 420–424. 27096365 10.1038/nature17946 PMC4873371

[B25] Kump, K.L., P.J. Bradbury, R.J. Wisser, E.S. Buckler, A.R. Belcher, M.A. Oropeza-Rosas, J.C. Zwonitzer, S. Kresovich, M.D. McMullen, D. Ware et al. (2011) Genome-wide association study of quantitative resistance to southern leaf blight in the maize nested association mapping population. Nat Genet 43: 163–168. 21217757 10.1038/ng.747

[B26] Kwart, D., D. Paquet, S. Teo and M. Tessier-Lavigne (2017) Precise and efficient scarless genome editing in stem cells using CORRECT. Nat Protoc 12: 329–354. 28102837 10.1038/nprot.2016.171

[B27] Li, Y., J. Zhu, H. Wu, C. Liu, C. Huang, J. Lan, Y. Zhao and C. Xie (2020) Precise base editing of non-allelic acetolactate synthase genes confers sulfonylurea herbicide resistance in maize. Crop J 8: 449–456.

[B28] Luo, W., R. Suzuki and R. Imai (2023) Precise *in planta* genome editing via homology-directed repair in wheat. Plant Biotechnol J 21: 668–670. 36529912 10.1111/pbi.13984 PMC10037140

[B29] Mali, P., L. Yang, K.M. Esvelt, J. Aach, M. Guell, J.E. DiCarlo, J.E. Norville and G.M. Church (2013) RNA-guided human genome engineering via Cas9. Science 339: 823–826. 23287722 10.1126/science.1232033 PMC3712628

[B30] Matres, J.M., J. Hilscher, A. Datta, V. Armario-Nájera, C. Baysal, W. He, X. Huang, C. Zhu, R. Valizadeh-Kamran, K.R. Trijatmiko et al. (2021) Genome editing in cereal crops: An overview. Transgenic Res 30: 461–498. 34263445 10.1007/s11248-021-00259-6 PMC8316241

[B31] Miki, D., W. Zhang, W. Zeng, Z. Feng and J.-K. Zhu (2018) CRISPR/Cas9-mediated gene targeting in *Arabidopsis* using sequential transformation. Nat Commun 9: 1967. 29773790 10.1038/s41467-018-04416-0 PMC5958078

[B32] Nagamine, A. and H. Ezura (2022) Genome editing for improving crop nutrition. Front Genome Ed 4: 850104. 35224538 10.3389/fgeed.2022.850104 PMC8864126

[B33] Nerkar, G., S. Devarumath, M. Purankar, A. Kumar, R. Valarmathi, R. Devarumath and C. Appunu (2022) Advances in crop breeding through precision genome editing. Front Genet 13: 880195. 35910205 10.3389/fgene.2022.880195 PMC9329802

[B34] Paquet, D., D. Kwart, A. Chen, A. Sproul, S. Jacob, S. Teo, K.M. Olsen, A. Gregg, S. Noggle and M. Tessier-Lavigne (2016) Efficient introduction of specific homozygous and heterozygous mutations using CRISPR/Cas9. Nature 533: 125–129. 27120160 10.1038/nature17664

[B35] Peiffer, J.A., M.C. Romay, M.A. Gore, S.A. Flint-Garcia, Z. Zhang, M.J. Millard, C.A.C. Gardner, M.D. McMullen, J.B. Holland, P.J. Bradbury et al. (2014) The genetic architecture of maize height. Genetics 196: 1337–1356. 24514905 10.1534/genetics.113.159152 PMC3982682

[B36] Poland, J.A., P.J. Bradbury, E.S. Buckler and R.J. Nelson (2011) Genome-wide nested association mapping of quantitative resistance to northern leaf blight in maize. Proc Natl Acad Sci USA 108: 6893–6898. 21482771 10.1073/pnas.1010894108 PMC3084105

[B37] Porteus, M. (2016) Genome editing: A new approach to human therapeutics. Annu Rev Pharmacol Toxicol 56: 163–190. 26566154 10.1146/annurev-pharmtox-010814-124454

[B38] Rao, S., Y. Yao and D.E. Bauer (2021) Editing GWAS: Experimental approaches to dissect and exploit disease-associated genetic variation. Genome Med 13: 41. 33691767 10.1186/s13073-021-00857-3 PMC7948363

[B39] Sarkar, A., K. Panati and V.R. Narala (2022) Code inside the codon: The role of synonymous mutations in regulating splicing machinery and its impact on disease. Mutat Res Rev Mutat Res 790: 108444. 36307006 10.1016/j.mrrev.2022.108444

[B40] Schaid, D.J., W. Chen and N.B. Larson (2018) From genome-wide associations to candidate causal variants by statistical fine-mapping. Nat Rev Genet 19: 491–504. 29844615 10.1038/s41576-018-0016-z PMC6050137

[B41] Schnable, P.S., D. Ware, R.S. Fulton, J.C. Stein, F. Wei, S. Pasternak, C. Liang, J. Zhang, L. Fulton, T.A. Graves et al. (2009) The B73 maize genome: Complexity, diversity, and dynamics. Science 326: 1112–1115. 19965430 10.1126/science.1178534

[B42] Svitashev, S., J.K. Young, C. Schwartz, H. Gao, S.C. Falco and A.M. Cigan (2015) Targeted mutagenesis, precise gene editing, and site-specific gene insertion in maize using Cas9 and guide RNA. Plant Physiol 169: 931–945. 26269544 10.1104/pp.15.00793 PMC4587463

[B43] Tam, V., N. Patel, M. Turcotte, Y. Bossé, G. Paré and D. Meyre (2019) Benefits and limitations of genome-wide association studies. Nat Rev Genet 20: 467–484. 31068683 10.1038/s41576-019-0127-1

[B44] Wallace, J.G., P.J. Bradbury, N. Zhang, Y. Gibon, M. Stitt and E.S. Buckler (2014) Association mapping across numerous traits reveals patterns of functional variation in maize. PLoS Genet 10: e1004845. 25474422 10.1371/journal.pgen.1004845 PMC4256217

[B45] Waltz, E. (2022) GABA-enriched tomato is first CRISPR-edited food to enter market. Nat Biotechnol 40: 9–11. 34907351 10.1038/d41587-021-00026-2

[B46] Wang, J.Y. and J.A. Doudna (2023) CRISPR technology: A decade of genome editing is only the beginning. Science 379: eadd8643. 36656942 10.1126/science.add8643

[B47] Xu, H., L. Zhang, K. Zhang and Y. Ran (2020) Progresses, challenges, and prospects of genome editing in soybean (*Glycine max*). Front Plant Sci 11: 571138. 33193504 10.3389/fpls.2020.571138 PMC7642200

[B48] Yasir, M., S. He, G. Sun, X. Geng, Z. Pan, W. Gong, Y. Jia and X. Du (2019) A genome-wide association study revealed key SNPs/genes associated with salinity stress tolerance in upland cotton. Genes 10: 829. 31640174 10.3390/genes10100829 PMC6826536

[B49] Zhang, W., R. Wang, D. Kong, F. Peng, M. Chen, W. Zeng, F. Giaume, S. He, H. Zhang, Z. Wang et al. (2023) Precise and heritable gene targeting in rice using a sequential transformation strategy. Cell Rep Methods 3: 100389. 36814841 10.1016/j.crmeth.2022.100389 PMC9939429

[B50] Zimin, A.V., D. Puiu, R. Hall, S. Kingan, B.J. Clavijo and S.L. Salzberg (2017) The first near-complete assembly of the hexaploid bread wheat genome, *Triticum aestivum*. Gigascience 6: gix097. 10.1093/gigascience/gix097 PMC5691383 29069494

